# Co-Design and Evaluation of a Gamified E-Resource About Healthcare Decarbonisation: A Study Protocol

**DOI:** 10.3390/nursrep15120447

**Published:** 2025-12-13

**Authors:** Nuala McLaughlin-Borlace, Stephanie Craig, Nuala Flood, Laura Steele, Tara Anderson, Sara Lynch, Jesús Sánchez-Martín, Rose Gallagher, Naomi Tutticci, Charlotte McArdle, Tracy Levett-Jones, Fadwa Al Halaiqa, Dalal Hammodi Halat, Norfadzilah Binti Ahmad, Gary Mitchell

**Affiliations:** 1 School of Nursing and Midwifery, Queen’s University Belfast, Belfast BT9 7BL, UK; s.craig@qub.ac.uk (S.C.); tanderson@qub.ac.uk (T.A.); gary.mitchell@qub.ac.uk (G.M.); 2 School of Natural and Built Environment, Queen’s University Belfast, Belfast BT9 5AG, UK; n.flood@qub.ac.uk; 3 Queen’s Business School, Queen’s University Belfast, Belfast BT9 5EE, UK; laura.steele@qub.ac.uk; 4 Estates Directorate, Queen’s University Belfast, Belfast BT7 1NN, UK; s.lynch@qub.ac.uk; 5 Facultad de Educación y Psicología, Universidad de Extremadura, 06006 Badajoz, Spain; jsanmar@unex.es; 6 Royal College of Nursing, London W1G 0RN, UK; rose.gallagher@rcn.org.uk; 7 School of Nursing and Midwifery, Griffith University, Brisbane 4111, Australia; n.tutticci@griffith.edu.au; 8 Faculty of Health Science, Ulster University, Northland Rd, Londonderry BT48 7JL, UK; mcardle-c30@ulster.ac.uk; 9 School of Nursing and Midwifery, University of Technology Sydney (UTS), Ultimo 2007, Australia; tracy.levett-jones@uts.edu.au; 10 Pre-Clinical Affairs, College of Nursing, Qatar University, Doha P.O. Box 2713, Qatar; f.alhalaiqa@qu.edu.qa; 11 QU Health Office of Assessment and Accreditation, QU Health, Qatar University, Doha P.O. Box 2713, Qatar; dhammoude@qu.edu.qa; 12 Faculty of Nursing, International Islamic University Malaysia, Kuantan Campus, Kuantan 25200, Pahang, Malaysia; fadzilah_hmd@iium.edu.my

**Keywords:** healthcare decarbonisation, sustainable healthcare, climate change, carbon emissions, health professions education, gamified learning, digital education, co-design

## Abstract

Climate change poses a major global health threat, with healthcare systems contributing substantially to global greenhouse gas emissions. Health professionals and students play an essential role in advancing sustainable practice, yet many lack the knowledge, skills, and confidence needed to address the environmental impacts of healthcare. This study aims to co-design and evaluate a gamified e-resource that enhances pre-registration health profession students’ knowledge, self-efficacy, and attitudes towards healthcare decarbonisation, while encouraging sustainable behaviour change. A sequential explanatory design will be employed in three phases: (1) a scoping review of the literature; (2) four co-design workshops with students (n = 20) followed by post-workshop focus groups using focused ethnography to explore co-design experiences; and (3) pre- and post-test questionnaires (n = 200) assessing knowledge, attitudes, self-efficacy, behaviours, willingness to act, and usability, followed by focus groups (n = 30) exploring behavioural changes after using the e-resource. The study will generate evidence on how a co-designed, gamified e-resources influence student learning and engagement with healthcare decarbonisation. Findings will inform the integration of sustainability and decarbonisation principles within education and support efforts to equip future health professionals with the competencies required for a low-carbon healthcare system.

## 1. Introduction

According to the World Health Organization, climate change is recognised as the greatest threat to global health of the 21st century, affecting population health, healthcare delivery, and the stability of healthcare systems worldwide [1]. Human activities, particularly fossil fuel combustion, industrial processes, and deforestation, have driven atmospheric carbon dioxide (CO_2_) concentrations to levels unprecedented in the past 800,000 years, with current measurements exceeding 420 parts per million [2,3]. If current trends persist, global temperatures could rise to an average of 3.2 °C by the year 2100, increasing the frequency and intensity of extreme weather events, exacerbating infectious disease transmission, and threatening food, water, and national security [4].

In response, international organisations including the United Nations (UN) and the World Health Organization (WHO) have called for systemic action to mitigate climate change and safeguard planetary health. The UN Sustainable Development Goals (SDGs), particularly SDG 13 (Climate Action) and SDG 3 (Good Health and Well-Being), emphasise the need to integrate environmental sustainability into health systems [5]. Similarly, the WHO advocates for climate-resilient and low-carbon health systems that both adapt to climate impacts and reduce their environmental footprint [6].

The healthcare sector itself contributes substantially to greenhouse gas (GHG) emissions, accounting for 4–5% of total emissions worldwide, a footprint comparable to the aviation industry [7,8]. Emissions are classified across internationally recognised categories into three scopes [9]; Scope 1 emissions are direct emissions generated by healthcare organisations themselves, such as on-site fuel combustion for heating, hospital vehicle fleets, and anaesthetic gases released during clinical procedures. Scope 2 emissions are indirect emissions associated with the generation of purchased energy, including electricity, steam, heating, and cooling used within healthcare facilities. Scope 3 emissions encompass all other indirect emissions across the healthcare supply chain, representing the largest proportion of the sector’s footprint. These include emissions from the manufacture, transport, and disposal of pharmaceuticals, medical devices, food, and equipment, as well as staff and patient travel and outsourced services [7,8,9]. Addressing all three scopes is therefore essential for effective healthcare decarbonisation, with Scope 3 posing the greatest challenge due to its complexity and dependence on upstream suppliers and procurement practices. Decarbonisation efforts in healthcare therefore require comprehensive strategies spanning infrastructure, procurement, models of care, and professional practices. At COP26, over 50 countries committed to implementing climate-resilient and low-carbon healthcare systems, highlighting the urgency of coordinated global action [10]. However, successful implementation depends not only on policy and infrastructural transformation but also on embedding sustainability into health professional education, preparing the workforce to deliver environmentally responsible care.

Health professionals are key to implementing sustainable practices, yet many report feeling underprepared, lacking the knowledge, confidence, or skills required [11]. Although frameworks such as the Global Consortium on Climate and Health Education (GCCHE) provide guidance, integration into pre-registration curricula remains inconsistent, fragmented, and often optional [12,13]. Educators also report insufficient training to teach sustainability and limited opportunities for students, despite global recognition of the need to implement core training on the impact of climate on health [14,15]. Without structured, evidence-based education, health professionals can struggle to implement sustainable practices effectively or advocate for decarbonisation within healthcare systems. Addressing these barriers through targeted educational interventions is critical to empowering the workforce and accelerating the adoption of sustainable healthcare practices.

Pre-registration health profession students represent the future healthcare workforce and are therefore a critical population for embedding sustainable practices early in professional development. Despite recognising the urgency of climate action and their responsibility as future practitioners, many students report feeling underprepared to apply sustainability principles in practice [11,16,17,18,19]. This reflects a gap in current pre-registration education, where sustainability content remains fragmented and inconsistently embedded. Strengthening self-efficacy at this formative stage is therefore crucial, as it underpins both motivation and the translation of knowledge and competence into behaviour [20]. Although gaps in sustainability knowledge persist, evidence indicates that knowledge alone is insufficient to drive behaviour change. Learners may demonstrate a strong understanding of sustainability concepts but this does not consistently translate into positive attitudes or behaviours; rather, attitudes exert a stronger influence on behavioural outcomes [21]. Therefore, this study prioritises enhancing attitudes, self-efficacy, and behaviours, alongside knowledge, to ensure that students are not only informed but also empowered and motivated to apply sustainability principles effectively within healthcare practice.

Digital platforms, gamified content, and case-based learning approaches are consistently reported as engaging, motivating, and effective for knowledge retention [22,23]. For example, a co-designed podcast with nursing and midwifery students significantly improved knowledge and perceived relevance of the Sustainable Development Goals (SDGs) and promoted subtle yet meaningful behavioural changes, including energy conservation, recycling, participation in health promotion activities, and influencing peers and family to adopt more sustainable practices. Participants also reported enhanced confidence and motivation to translate their knowledge into everyday actions, demonstrating the potential for digital resources to bridge the gap between learning and practice [24].

Gamified education, defined as the integration of game design elements into learning experiences to enhance academic performance, has emerged as a highly effective pedagogical approach in health professions education and increased rapidly in recent years [25,26]. Digital gamified resources have been shown to enhance knowledge acquisition, increase performance, improve self-efficacy, and overcome traditional barriers such as cost, accessibility, and time constraints [27]. Gamification in health profession education is often as effective as, and in many cases more effective than, traditional methods for improving knowledge, skills, and satisfaction, though the evidence remains low-quality and calls for more rigorous, theory-driven research [28]. Examples of successful gamified interventions include training on dementia care, COVID-19 management, and other complex clinical topics [29,30].

Despite these advances, no gamified e-resource currently targets healthcare decarbonisation, particularly one co-designed with students [31,32]. Co-design empowers learners to shape content, ensuring relevance and usability. This can enhance self-efficacy by giving students confidence in applying what they learn and supporting the translation of knowledge into behaviours [33]. Developing such an e-resource provides an important opportunity to address gaps in sustainability education and prepare future health professionals for environmentally responsible practice.

Accordingly, the aim of this study is to co-design and evaluate a gamified e-resource on healthcare decarbonisation for pre-registration health profession students in Northern Ireland (NI).

Objectives:

- To conduct a scoping review of existing educational resources on healthcare decarbonisation for pre-registration health profession students.
- To co-design a gamified digital learning e-resource on healthcare decarbonisation with health profession students using an Accelerated Experience-Based Co-Design approach.
- To evaluate the impact of the gamified digital learning e-resource on students’ attitudes, self-efficacy, behaviour change, and acceptability.

International Observer Panel (IOP)

The intervention developed in this study is intended to support healthcare decarbonisation education within the participating institution and, if successful, could also be adapted and scaled nationally and internationally. An International Observer Panel (IOP) has been established to support study development and facilitate future scalability of the e-resource.

The IOP includes representatives and experts in healthcare decarbonisation, climate change, sustainability, planetary health, co-design, and healthcare education. Members include seven academics and two practice and policy representatives across multiple countries including Australia, Spain, Qatar, Malaysia, and the UK (SL, JSM, RG, CMC, TLJ, NT, FA, DH, NBA). The panel will meet biannually via Microsoft Teams, aligned with study milestones, for example, literature review development, co-design, and differentiation. Ongoing communication will also be maintained via email. The IOP acts as a group of strategic partners, providing high-level guidance and expert insight across all phases of the study to ensure international relevance, methodological rigour, and alignment with best practice in sustainability education. IOP members will be able to provide feedback in relation to the use and potential adaption of the e-resource within their respective countries, promoting and supporting international dissemination of the study. Members of the IOP provide advisory international expertise across the project and will be acknowledged for their contribution to study development.

## 2. Materials and Methods

### 2.1. Methods

This study employs a sequential explanatory mixed-methods research (MMR) design, integrating quantitative and qualitative approaches to generate a comprehensive understanding of healthcare decarbonisation education. The MMR framework enables deliberate combination of multiple data sources and analytical strategies, yielding understanding not achievable through a single method [34]. This approach allows findings from one stage to inform subsequent data collection and analysis, ensuring iterative refinement and integration of evidence, user perspectives, and contextual factors.

Results from the scoping review will identify gaps, priorities, and effective strategies in healthcare decarbonisation education, directly shaping the focus, content, and design of the accelerated experience-based co-design (AEBCD) workshops. Findings from the co-design process, including qualitative feedback and field observations, will guide the development of the gamified e-resource, ensuring it is contextually relevant, learner-centred, and responsive to stakeholder needs. Finally, the mixed-methods evaluation of the e-resource will provide quantitative data relating to changes in knowledge, attitudes, self-efficacy, willingness to act, behaviours, and affective response, alongside qualitative exploration into behaviour, usability, and learning experiences, which can be used to refine the intervention and inform future iterations. This phased integration enhances both the relevance and the rigour of the study. At the time of manuscript submission, Phase 1 had been completed. Phase 2 and Phase 3, involving empirical data collection, co-design, delivery, and evaluation of the gamified e-resource, is fully prospective.

### 2.2. Study Design

**Figure 1:**
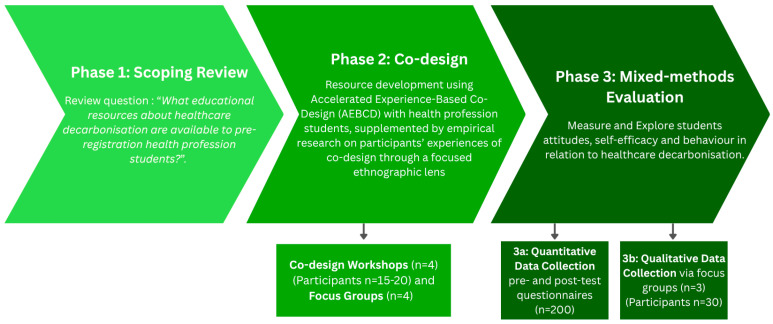
Study design graphic.

The study will follow three main phases: (1) a scoping review of the literature; (2) co-design phase; and (3) mixed-methods evaluation (Figure 1).

Phase 1: Scoping Review

A scoping review was conducted to synthesise emerging evidence on healthcare decarbonisation education within health profession training. The review specifically examined educational resources addressing healthcare decarbonisation for pre-registration health profession students, including those in nursing, midwifery, medicine, pharmacy, dentistry, and allied health education. A scoping review is appropriate for mapping this breadth of evidence, clarifying concepts, and identifying knowledge gaps to inform the co-design phase of this project (Phase 2) [35]. The review followed the Joanna Briggs Institute methodology and the PRISMA-ScR framework [36,37]. A review protocol has been registered with the Open Science Framework (OSF) (https://doi.org/10.17605/OSF.IO/2W7UP, 14 April 2025).

Previous reviews have explored climate change and sustainability education in the health professions. They have highlighted facilitators including environmental behaviours, self-directed learning, and educator engagement, alongside barriers such as curriculum overload and limited faculty expertise [38,39]. Evidence also points to positive impacts across multiple professions; however, no single pedagogical approach has emerged as clearly superior, and the need for scalable, practice-ready curricula has been emphasised [31,40]. Interprofessional education initiatives have been reported, yet most interventions remain discipline-specific with limited collaboration [41,42], while increasing awareness among health professionals is constrained by institutional challenges [43]. Core environmental competencies, such as systems thinking and resource stewardship, have been mapped to wider healthcare standards [44].

While sustainability education is expanding, knowledge alone does not guarantee sustainable practice. Translating knowledge into action relies on broader influences including motivation, attitudes, self-efficacy, and engagement [45]. Importantly, no review has specifically examined healthcare decarbonisation, an active, practice-oriented response to climate change, leaving a gap in understanding how educational interventions can prepare pre-registration health profession students to apply sustainability principles effectively. Together, these reviews demonstrate progress in embedding sustainability principles, but none explicitly address healthcare decarbonisation as an urgent and under-explored area. This scoping review mapped current educational resources on healthcare decarbonisation for pre-registration health profession students, identified gaps and opportunities for curriculum development, and provided the evidence base for the subsequent co-design of an educational intervention (phase 2). The IOP contributed to feedback during the write up stage and supported refinement of key findings, barriers, and touch points, which directly informed the selection of the trigger materials used in phase 2.

Eligibility Criteria

The eligibility criteria were guided by the Population, Concept, and Context (PCC) framework [44]. The population comprised pre-registration health profession students, including those studying nursing, medicine, pharmacy, dentistry, and allied health disciplines. These students represent the future healthcare workforce, who collectively will deliver the majority of clinical care and are therefore key contributors to healthcare-related carbon emissions. Post-graduate, PhD, and registered health professionals were excluded to maintain focus on early-stage curricula. The concept of interest was healthcare decarbonisation education, encompassing all relevant forms of interventions (e.g., digital games, workshops, or face-to-face teaching). The context included any setting where such education is delivered. Both qualitative and quantitative studies published in English were included, with no geographical or date restrictions. Although grey literature is often included in scoping reviews, it was not considered. The review focused exclusively on peer-reviewed evidence to ensure greater robustness and comparability with other studies.

Data Collection

An initial limited search of CINAHL informed development of the search strategy, which was adapted for six databases: CINAHL, PsycINFO, MEDLINE, GreenFILE, Scopus, and Web of Science. Reference lists of included articles were also screened. All citations were imported into EndNote, then transferred to Covidence (https://www.covidence.org/ accessed on 1 May 2025) for screening and data management. Duplicates were removed. Title/abstract and full-text screening was carried out independently by at least two reviewers (NM and SC), with disagreements resolved by discussion or consultation with a third reviewer. Reasons for exclusion at the full-text stage were documented.

Data extraction utilised the Joanna Briggs Institute Template for Source of Evidence Details, Characteristics and Results Extraction Instrument [46]. The tool was piloted and adapted as necessary. Extracted information included study characteristics (author, year, country), participants, type of intervention, context, methods, and key findings. One reviewer (NM) extracted relevant data, and another (SC) independently verified accuracy.

Measures

The review extracted study identifiers (author, year, country/setting), participant characteristics and sample size, type of educational resource, intervention details, context, and methods and reported findings relevant to healthcare decarbonisation learning outcomes.

Data Analysis

The study selection process was reported using a PRISMA-ScR flow diagram [30]. Extracted data was presented in tabular format summarising study characteristics (author, year, country, aims, population, methods, and findings). A narrative synthesis explained how results address the review question and objectives. Content analysis was used to map key patterns, and thematic analysis will identify broader themes, overlaps, and gaps in the literature [47,48].

Phase 2: Co-Design

Phase 2 will involve four face-to-face co-design workshops conducted over two months with a total of 15–20 participants overall, including pre-registration health profession students, academics, PPI members, IOP representatives, and sustainability experts. The number of workshops and participants has been chosen to balance diversity of perspectives with feasibility. The face-to-face format is intended to foster richer interaction, relationship-building, and in-depth iterative feedback. Conducting workshops within a two-month period ensures momentum is maintained while allowing sufficient time for reflection and preparation between sessions.

To reduce the time and resource demands of traditional Experience-Based Co-Design (EBCD), an Accelerated Experience-Based Co-Design (AEBCD) model will be used [49,50]. AEBCD leverages pre-existing narrative resources, such as filmed interviews from Health Talk Online and similar repositories, which include patient and professional accounts of experiences with sustainability initiatives and challenges in healthcare practice. These materials will be adapted into trigger resources (e.g., vignettes, short videos, infographics) to stimulate reflection, discussion, and idea generation, thereby reducing the need for extensive new data collection while retaining the narrative-driven impact central to EBCD. Evidence from healthcare quality improvement demonstrates AEBCD’s feasibility and effectiveness, showing that structured collaborative forums foster mutual understanding and generate meaningful exchange among stakeholders [51,52]. Trigger materials will be selected based on (1) relevance to healthcare decarbonisation practices, (2) authenticity of the narrative, (3) representation of commonly reported challenges or enablers identified in Phase 1, and (4) applicability to pre-registration health profession students. Materials will be adapted into short vignettes, videos, and infographics to be included within workshop 1’s capacity building presentation. Members of the research team and the International Observer Panel will validate the final trigger materials to ensure accuracy, clarity, and pedagogical relevance.

The AEBCD process will follow four stages. First, experiences will be synthesised and key touchpoints identified, drawing on the scoping review (phase 1) and open-access narratives to highlight recurring barriers and enablers, including knowledge gaps, decision-making barriers, and workflow challenges. Second, trigger materials will be developed into vignettes, short videos, and infographics to guide participant reflection and discussion. Third, participants will engage in co-design workshops to provide feedback on these materials, prioritise learning outcomes, and collaboratively design and prototype the e-resource using AEBCD principles alongside a focused ethnography approach. Outputs from Phase 2, including prioritised learning outcomes, feedback, user-identified touch points, and preferred gamification features, will directly inform the e-resource development. These outputs will determine the content, structure, delivery, and gamified elements of the e-resource. Fourth, a synthesised prototype will be presented at a final review event for participants to confirm features, map implementation pathways, and co-develop evaluation metrics. Prototypes will be shared with the IOP for focused expert feedback on content and usability, including input on proposed evaluation metrics (phase 3).

Focused ethnography will be embedded within the AEBCD workshops to capture the social, cultural, and contextual dynamics of the co-design process [53,54]. Immediately following each workshop, focus groups will explore participants’ lived experiences of the process, providing empirical data on effective elements, challenges, and potential refinement [55]. All workshops and focus groups will be conducted face-to-face at the authors’ home institution in NI.

Sampling Procedures

All pre-registration students enrolled in their chosen health profession courses (nursing, midwifery, medicine, pharmacy, dentistry) will be eligible to participate in Phases 2 and 3, following the inclusion and exclusion criteria (Table 1 and Table 2). Participants will be recruited via convenience sampling, enabling efficient engagement across disciplines and stages of training. While this approach may limit generalisability, it captures diverse perspectives relevant to the co-design and evaluation of the e-resource. Recruitment will seek representation across courses and demographics; however, participation is voluntary, and students can choose whether to take part in the study.

Participants and Recruitment

Participants will include students currently enrolled in pre-registration health profession programmes, specifically nursing (adult; learning disability; children and young people; mental health), midwifery, medicine, pharmacy, and dentistry. Recruitment will be facilitated through the Directors of Education at the authors’ home institution in NI, who will act as gatekeepers by distributing an email invitation to all eligible students. The gatekeepers will not be directly involved with the research study. This email will include a participant information sheet and the research team’s contact details. Interested students will be asked to contact the research team directly to express interest in joining the co-design workshops. Prior to participation, each student must complete a written consent form confirming they have read the participant information sheet. Students will be informed that participation is voluntary and will not affect their academic standing or grades.

Participants may indicate interest in participating in Phase 2 focus groups by ticking a box at the bottom of the co-design workshop written consent form. These participants will be invited to participate in the focus group interviews at the end of each of the four co-design workshops. A separate participant information sheet and written consent from will be provided. Participation in the focus groups will remain voluntary, regardless of workshop involvement. Full inclusion and exclusion criteria for phase 2 can be seen in Table 1.

Data Collection, Measures, and Analysis:

During the co-design workshops, participants will complete demographic questionnaires (age, sex, gender, ethnicity, field/year of study). Researcher field notes will capture contextual factors, group dynamics, and reflections as part of the focused ethnography. Immediately after each workshop, follow-up focus groups will be conducted to explore participants’ experiences of the process. These will be audio-recorded, transcribed verbatim, and anonymised. Data will be stored securely and retained for five years in accordance with institutional policy. Quantitative demographic data will be analysed descriptively. Qualitative data, including field notes, focus group transcripts, and open reflections, will be analysed iteratively. Rapid Qualitative Inquiry (RQI) will be used to identify and refine emerging themes from the researcher field notes. Reflexive thematic analysis following Braun and Clarke’s six-phase framework [48] will be applied to the focus group data to generate deeper themes. Data management and coding will be supported by NVivo 14, ensuring transparency and rigour in the analytic process.

Phase 3: Mixed-Methods Evaluation

The third phase will comprise a mixed-methods evaluation of the e-resource, delivered to pre-registration health profession students via the institutional Learning Management System (LMS). Evaluation and implementation strategies will be informed via student feedback and preferences collected during phase 2 co-design. This phase aims to assess four primary study outcomes: attitudes, self-efficacy, behaviours, and usability. Evaluation will occur in two parts. Phase 3a involves pre- and post-test questionnaires to measure associated changes in student (n = 200) knowledge, attitudes, self-efficacy, behaviour, willingness to act, affective responses, and usability. Phase 3b consists of three focus group interviews with participating students (n = 30), designed to explore perceived behavioural changes and acceptability and to provide further interpretation of associated quantitative findings. The e-resource will be delivered asynchronously and made available to students for a four-week period via a generic healthcare module within each discipline. Engagement will be voluntary, allowing students to access the e-resource at their own pace. To ensure that evaluation findings reflect the impact of the intervention, the e-resource will be delivered independently and will not form part of any other educational content on decarbonisation, sustainability, or climate change. This approach allows students to engage flexibly alongside academic and clinical commitments, while also ensuring feasibility for the research team in relation to data collection and analysis. To ensure transparency, rigour, and high-quality reporting, the study will be guided by the Good Reporting of a Mixed Methods Study (GRAMMS) framework, thereby adhering to recognised best practice in mixed-methods research [56].

Participants and Recruitment

Phase 3a: Approximately n = 200 healthcare students will be recruited from nursing, midwifery, medicine, dentistry, and pharmacy at the authors home institution in NI. A sample size of n = 200 was selected for the quantitative evaluation phase as this aligns with comparable intervention studies in health profession education [57,58,59]. As this study is focused on evaluating acceptability rather than testing intervention efficacy, a formal power calculation was not conducted. Acceptability studies commonly rely on pragmatic sample estimates; therefore, a target of approximately n ≈ 200 was selected to obtain a sufficiently diverse sample across health profession programmes and to support stable descriptive and exploratory analysis. With permission of the Director of Education (from the Schools of Medicine, Nursing and Midwifery, Pharmacy, and Dentistry) students will be invited to engage with the e-resource. All pre-registered health profession students will be contacted via email by their Director of Education to inform them about this evaluation. They will act as a gatekeeper to the study. The email will contain the participant information sheet and contact details of the research team. Students will be emailed once about this project, and the email and information sheet will be placed on the module page where students will access the e-resource. Each student will need to complete a digital participant consent form and confirm they have read the participant information sheet prior to taking part in the study. If students have any questions prior to enrolling in the study they will be directed to contact the research team.

Phase 3b: Students will be invited to email an expression of interest to the chief investigator to participate in the focus group interviews. A purposive sample size of n = 30 was chosen to facilitate approx. 3–4 focus groups with 8–10 participants in each, ensuring diversity of views between professions while achieving data saturation. The sample will be mixed in terms of programme of study to capture a broad range of perspectives. Prior to participation they will receive an information sheet and will be required to provide written consent on the day of the focus group interview. Participants must have fully engaged in phase 3a to be eligible to participate in phase 3b focus groups. Students will be informed that phase 3b focus groups are voluntary. Full inclusion exclusion criteria for phase 3 can be seen in Table 2.

Data Collection

Phase 3a: After accessing the link to the e-resource, students will be automatically directed to the participant information sheet and provided an opportunity to consent to the e-resource testing. Students who wish to take part in the evaluation will click on the pre- and post-weblinks (located on the module page where the e-resource may be accessed) to be taken to each MS Form both before and after engaging with the e-resource.

Phase 3b: All focus groups will be digitally audio-recorded only. Audio will be fully transcribed verbatim by the research team. Management of focus group data for Phase 3b will be conducted by the research team, following the same procedures used for the Phase 2 focus group interviews.

Measures

Phase 3a: A pre- and post-test design survey will be available within the e-resource via a MS Forms link. The pre-test will include a demographic questionnaire (age, sex, gender, ethnicity, field/year of study) and a 36-item Sustainability Perception Questionnaire [60]. The Sustainability Perception Questionnaire is a validated instrument designed to assess university students’ perceptions of sustainability. It encompasses six dimensions: knowledge, attitude, willingness to act, behaviour, self-efficacy, and affective responses. The questionnaire employs a mixed-methods approach, combining 6-point Likert scale items with closed and open-ended questions. Confirmatory factor analysis confirmed the model’s good fit, and internal reliability coefficients ranged from 0.737 to 0.909, confirming a high level of internal reliability. This tool offers a reliable and valid means for evaluating sustainability perceptions among university students, facilitating the development of targeted educational strategies. For this study, the primary outcomes of interest are attitudes, self-efficacy, and behaviour, reflecting the main focus of the intervention on enhancing students’ capacity to translate knowledge into sustainable practice. The remaining dimensions, knowledge, willingness to act, and affective responses, will be considered secondary outcomes to provide additional context and understanding into the broader impact of the e-resource. Example items relating to healthcare decarbonisation include (17.) I must change my lifestyle to create less waste (for example, throwing away less food or not wasting certain products), (18.) I would participate in activities that make the faculty more sustainable, (20.) I am willing to change my consumption style to be more sustainable (energy, food, transportation, etc.), and (23.) I always separate waste, such as plastics, glass, etc., from food scraps, before depositing them in the recycling bins. The post-test will repeat the pre-test questionnaires in addition to the 12-item User Engagement Scale Short Form (UES-SF) and four open-text questions [61]. The UES-SF measures users’ overall engagement with a digital application across four domains: focused attention, perceived usability, aesthetic appeal, and reward. The open-text questions will consist of three questions for evaluation of the Healthcare Decarbonisation Gamified E-resource including likes, dislikes, and ideas for further dissemination/testing, and one question asking whether the participant had any prior exposure with any previous sustainability or climate change education resources.

Phase 3b: A focus group guide will be developed specifically for this study, based on the literature review, the co-design process, and feedback from the International Observer Panel (IOP). Focus groups will be moderated by two members of the research team. Focus groups will be used to ask students to discuss any perceived changes in behaviour, discuss any changes in outcomes from quantitative results, explore acceptability of the e-resource, and share any suggestions for the future. The questions will provide a guide for the focus group but will also assist participant discussion in real time. Researcher-generated knowledge and behaviour items will be developed as no validated instruments currently exist that capture the short-term learning outcomes and proximal behavioural intentions relevant to healthcare decarbonisation education. These items will be informed by Phase 1 findings and Phase 2 co-design outputs. To strengthen validity, items will undergo face-validity testing with approximately 20 students (10% of the target sample), assessing clarity, relevance, and ease of understanding. Feedback from this pilot will be used to refine items prior to full deployment.

Data Analysis

Phase 3a:

Quantitative data from Phase 3a will be analysed using descriptive and inferential statistics. Descriptive analyses will summarise baseline characteristics and distributions, and assumptions of normality and homogeneity of variance will be tested prior to inferential statistics. Paired *t*-tests will be conducted to examine pre- and post-intervention differences in attitudes, self-efficacy, and behaviours. Correlation analyses will be undertaken using Pearson’s correlation for normally distributed continuous data and Spearman’s rank correlation for ordinal or non-parametric data to assess relationships between baseline variables and sustainability-related behaviours. Effect sizes (e.g., Cohen’s d) will be reported to indicate magnitude of observed changes. Multivariate analysis of variance (MANOVA) will be applied to explore differences between groups, such as professional discipline or prior exposure to sustainability education. Missing data will be assessed for extent and pattern and managed using listwise deletion when minimal or multiple imputation when substantial to preserve statistical power. These procedures will provide an overall measure of intervention impact as well as a look into variation across subgroups. As the study uses a single-group pre- and post-test design, findings will be interpreted as changes associated with the e-resource and not as causal effects.

Phase 3b:

Qualitative data from Phase 3b, including focus group transcripts and open-text survey responses will be analysed using reflexive thematic analysis following Braun and Clarke’s six-phase framework [48]. NVivo 14 will be used to support data management and coding. Reflexivity will be maintained via a researcher journal, team discussions of preconceptions and assumptions, and independent verification of transcripts. The IOP will provide iterative feedback through coding and theme development to ensure clarity, coherence, and relevance, thereby enhancing analytic rigour and reducing the risk of individual bias.

Data Integration

Mixed-methods integration will take place during the interpretation of results. Quantitative findings on associated changes in self-efficacy, attitudes, behaviours, and other secondary outcomes will be considered alongside qualitative results related to user experience, usability, and the perceived application of learning. Integration will be carried out using a triangulation approach, comparing and contrasting findings across data strands to identify convergence, complementarity, or divergence. This integrated approach will allow for a comprehensive understanding of the impact of the e-resource. It will also enable interpretation to be structured in relation to Kirkpatrick’s evaluation model by aligning learner reactions with qualitative data, knowledge, and confidence gains with quantitative results and behavioural application with evidence drawn from both strands [62,63].

Underpinning Framework

The evaluation of this study is informed by Kirkpatrick’s four-level model of training evaluation [62,63]. This model provides a structured approach for understanding not only immediate learner reactions but also knowledge gains, behavioural application, and longer-term outcomes. In this protocol, quantitative measures (e.g., pre- and post-test surveys of knowledge, attitudes, and self-efficacy) align primarily with Level 2 (Learning) and provide baseline evidence of change over time. Qualitative data from focus groups and ethnographic observations complement this by exploring Levels 1 (Reaction) and 3 (Behaviour), capturing students’ experiences of using the e-resource, their engagement with the co-design process, and perceived translation of learning into practice. This multi-level mapping will allow the study to demonstrate not only whether the intervention is effective but also how and why it influences student learning and behaviour. While Level 4 (results), long-term institutional outcomes, is beyond the scope of this study, the model nonetheless offers a useful integrative lens, ensuring that mixed-methods findings are interpreted along a continuum from reaction to behavioural change. In doing so, Kirkpatrick’s model helps to bridge quantitative and qualitative evidence, supporting a theoretical understanding of how a gamified e-resource may influence healthcare decarbonisation education.

### 2.3. Reflexivity

Reflexive thematic analysis will be used to analyse qualitative data from phases 2 and 3b. Reflexivity, the recognition of how a researcher’s background and prior assumptions may influence the research process, is a key component of Braun and Clarke’s six-phase framework for reflexive thematic analysis [48]. The six steps are (1) familiarisation with the data, (2) generating initial codes, (3) developing themes, (4) reviewing themes, (5) defining and naming themes, and (6) writing up the analysis. To enhance reflexivity, the research team will discuss preconceptions and assumptions prior to data analysis, transcriptions will be verified by another researcher, and all themes will be collaboratively composed by the research team. A researcher reflexive journal will be maintained throughout the analysis process, documenting reflections on initial impressions, coding decisions, and evolving interpretations. This journal will allow the researcher to critically consider how personal perspectives may shape theme development and ensure transparency in analytic decision-making. Additionally, the study IOP will provide iterative input throughout the analysis. During familiarisation and coding, the IOP will review initial codes and provide guidance on clarity and relevance. In the theme development and review stages, IOP members will offer critical feedback on the coherence, distinctiveness, and applicability of emerging themes. The IOP will also advise on the final definition, naming, and reporting of themes, ensuring that interpretations are both credible and grounded in broader professional and disciplinary perspectives. By combining a reflexive journal with IOP input at each stage of Braun and Clarke’s framework, this approach strengthens analytic rigour, mitigates individual bias, and ensures that findings are both transparent and informed by IOP perspectives.

### 2.4. Ethics and Governance

This study will be conducted in accordance with Ethical Committee guidelines and the Declaration of Helsinki [64]. The study received ethical approval from the Faculty of Medicine, Health and Life Sciences Research Ethics Committee at Queen’s University Belfast on the 26 June 2025 for Phase 2: co-design (Reference: MHLS 25_96) and on the 3rd September 2025 for Phase 3: evaluation (Reference: MHLS 25_131).

Written informed consent will be obtained from all participants prior to involvement in any phase of the study. Participants will receive a participant information sheet (PIS) specific to each phase and must complete a written consent form before participation. Access to and recruitment of students will be facilitated via a gatekeeper, the Director of Education, to ensure appropriate communication and coordination. Contact details for the Lead Researcher, Chief Investigator, and University Research Ethics Office will be provided to allow participants to ask questions or raise concerns. Participation is entirely voluntary, and participants will have the right to withdraw at any point without consequence. Given that student participants will be co-designing alongside faculty researchers, the study acknowledges potential power imbalances. To mitigate this, facilitation will emphasise mutual respect, psychological safety, and equitable contribution. Student voices will be explicitly valued as experiential expertise, and co-design sessions will be structured to promote inclusivity and shared decision-making. The well-being of both the participants and the researcher will be actively considered throughout the study.

## 3. Discussion

This study outlines a mixed-methods approach to the co-design and evaluation of a gamified e-resource aimed at strengthening health profession students’ attitudes, self-efficacy, and behaviours in relation to healthcare decarbonisation. It responds to a critical gap in current educational provision, where sustainability, climate change, and decarbonisation remain insufficiently represented despite increasing calls for integration [65,66,67]. Addressing this deficit is urgent given healthcare’s significant environmental footprint and the recognition of climate change as a global public health crisis [1].

Quantitative findings will provide evidence of changes in students’ knowledge, attitudes, and self-efficacy, while also identifying variation across subgroups such as discipline or prior exposure to sustainability content. Qualitative findings will add depth by capturing students’ experiences of co-design, perceptions of the e-resource, and reported behavioural application. Considered together, these strands will offer complementary perspectives: quantitative data will measure the extent of change, while qualitative findings will help explain how and why these changes occur.

The asynchronous design of the e-resource is expected to provide flexibility and learner autonomy, enabling students to engage at their own pace despite the competing demands of health profession programmes. This approach is particularly relevant in interprofessional education, where coordinating schedules across disciplines is challenging. Asynchronous learning may not fully develop collaborative skills; however, integrating interactive or synchronous elements alongside the e-resource could enhance team functioning and leadership development [68,69,70].

The recognised absence of structured, interprofessional educational resources on climate change and decarbonisation highlights the importance of this work [71,72]. By engaging students directly in co-design, the study will explore how contemporary digital learning tools can be shaped to support sustainability mindsets, peer learning, and interprofessional collaboration [73]. Recent work has further demonstrated the value of student co-design in developing digital educational resources, emphasising authentic participation, collaboration with academic teams, and the broader benefits for health education and practice [74]. Furthermore, input from the IOP will ensure that the resource is aligned with current professional standards and educational priorities, enhancing credibility and potential uptake via international dissemination in future. A limitation of this study is that it is being conducted within a single institution in Northern Ireland, which may affect generalisability; however, the involvement of the IOP aims to enhance transferability to other national and international contexts.

Ultimately, this study seeks to provide a practical entry point for including sustainability and decarbonisation more systematically across health curricula. By fostering interdisciplinary reflection and offering a resource grounded in both evidence and learner perspectives, it aspires to contribute to a broader cultural shift in health profession education where sustainability is positioned as a core element of ethical and evidence-based practice, rather than an optional addition. Future research will be needed to assess the longer-term impact of the e-resource on professional practice and to explore its scalability across diverse educational and contextual settings.

## Figures and Tables

**Table 1:** Phase 2 inclusion and exclusion criteria.

Inclusion	Exclusion
Students currently enrolled in a pre-registration health profession course (nursing (adult; learning disability; children and young people; mental health), midwifery, medicine, pharmacy, dentistry).	Post-graduate, registered, or PhD students. Students enrolled at another university in Northern Ireland (NI)
Consented to take part in study.	Students who did not consent to be a part of the study.

**Table 2:** Phase 3 inclusion and exclusion criteria.

Inclusion	Exclusion
Students currently enrolled in a pre-registration health profession course (Nursing (Adult; Learning Disability; Children and Young People; Mental Health), Midwifery Medicine, Pharmacy, Dentistry).	Post-graduate registered or PhD students. Students enrolled at another university in Northern Ireland (NI)
Consented to take part in the study.	Students who have not consented to be a part of the study.
Completed the healthcare decarbonisation e-resource.	Students who have not completed the healthcare decarbonisation e-resource.

## Data Availability

No data are available as this manuscript presents a study protocol.

## Public Involvement Statement

Members of the International Observer Panel (IOP), representing public, academic and practice stakeholders, contributed to the development of this protocol.

## Guidelines and Standards Statement

This protocol has been developed in accordance with established guidelines and reporting standards. The scoping review component will follow the PRISMA Extension for Scoping Reviews (PRISMA-ScR) (https://www.prisma-statement.org/scoping accessed on 1 May 2025). Qualitative elements, including co-design workshops and focus groups, will be reported in line with the Consolidated Criteria for Reporting Qualitative Research (COREQ) (https://www.equator-network.org/reporting-guidelines/coreq accessed on 25 April 2025). The mixed-methods evaluation is informed by the Transparent Reporting of Evaluations with Nonrandomized Designs (TREND) (https://www.equator-network.org/reporting-guidelines/improving-the-reporting-quality-of-nonrandomized-evaluations-of-behavioral-and-public-health-interventions-the-trend-statement accessed on 25 April 2025) guidelines.

## Abbreviations

The following abbreviations are used in this manuscript:

WHO	The World Health Organisation
UN	United Nations
NI	Northern Ireland
IOP	International Observer Panel
MMR	Mixed Methods Research
AEBCD	Accelerated Experience Based Co-Design
OSF	Open Science Framework
PCC	Population Concept Context
EBCD	Experience Based Co-Design
RQI	Rapid Qualitative Inquiry
UES	User Engagement Scale
MANOVA	Multivariate Analysis of Variance
PIS	Participant Information Sheet
IPE	Interprofessional Education
